# Hitting the Bull’s-Eye: Mesothelin’s Role as a Biomarker and Therapeutic Target for Malignant Pleural Mesothelioma

**DOI:** 10.3390/cancers13163932

**Published:** 2021-08-04

**Authors:** Dannel Yeo, Laura Castelletti, Nico van Zandwijk, John E. J. Rasko

**Affiliations:** 1 Li Ka Shing Cell & Gene Therapy Program, The University of Sydney, Camperdown, NSW 2050, Australia; dannel.yeo@sydney.edu.au (D.Y.); laura.castelletti@sydney.edu.au (L.C.); 2 Faculty of Medicine and Health, The University of Sydney, Camperdown, NSW 2050, Australia; nico.vanzandwijk@sydney.edu.au; 3 Cell and Molecular Therapies, Royal Prince Alfred Hospital, Sydney Local Health District (SLHD), Camperdown, NSW 2050, Australia; 4 Concord Repatriation General Hospital, Sydney Local Health District (SLHD), Concord, NSW 2139, Australia; 5 Gene and Stem Cell Therapy Program, Centenary Institute, The University of Sydney, Camperdown, NSW 2050, Australia

**Keywords:** cancer, malignant mesothelioma, malignant pleural mesothelioma, mesothelin, biomarker, therapeutic target, immunotherapy, CAR T cells

## Abstract

**Simple Summary:**

Mesothelioma is a deadly disease with a dismal prognosis. Since its discovery, mesothelin, a cell surface protein, has been a promising biomarker and therapeutic target due to its overexpression in mesothelioma and limited expression in normal cells. This review summarizes the clinical studies that have examined mesothelin as a biomarker and therapeutic target in mesothelioma and explores future perspectives in its role to improve patient management.

**Abstract:**

Malignant pleural mesothelioma (MPM) is an aggressive cancer with limited treatment options and poor prognosis. MPM originates from the mesothelial lining of the pleura. Mesothelin (MSLN) is a glycoprotein expressed at low levels in normal tissues and at high levels in MPM. Many other solid cancers overexpress MSLN, and this is associated with worse survival rates. However, this association has not been found in MPM, and the exact biological role of MSLN in MPM requires further exploration. Here, we discuss the current research on the diagnostic and prognostic value of MSLN in MPM patients. Furthermore, MSLN has become an attractive immunotherapy target in MPM, where better treatment strategies are urgently needed. Several MSLN-targeted monoclonal antibodies, antibody–drug conjugates, immunotoxins, cancer vaccines, and cellular therapies have been tested in the clinical setting. The biological rationale underpinning MSLN-targeted immunotherapies and their potential to improve MPM patient outcomes are reviewed.

## 1. Introduction

Mesothelioma is a rare malignancy arising from mesothelial cells lining the pleura, peritoneum, pericardium, and tunica vaginalis. Globally, it is estimated that 38,000–43,000 deaths per year are attributed to malignant mesothelioma, and the overall 5-year survival rate is less than 10% [1]. The majority (>80%) of malignant mesothelioma cases occur in the pleura, the serous membrane that lines the wall of the thoracic cavity and the surface of the lung, and this type is termed malignant pleural mesothelioma (MPM). MPM is predominantly diagnosed in individuals previously exposed to asbestos, and there is usually a long latency between asbestos exposure and diagnosis. There are prominent differences in MPM incidence reported from different countries worldwide. Incidence varies from 7 per million (Japan) to 40 per million (Australia) inhabitants per year. In Europe, the incidence is around 20 per million. The worldwide MPM incidence is difficult to determine as the disease is underreported in several countries [2,3].

MPM is resistant or quickly develops resistance to available therapies, invariably representing a fatal diagnosis. The early diagnosis of MPM is notoriously difficult from both clinical and pathological perspectives. Patients suspected of MPM often undergo multiple medical investigations without a definitive diagnosis. Therefore, biomarkers for (early) diagnosis, the estimation of prognosis, and treatment outcome prediction have received prominent attention [4,5,6]. Notwithstanding the limited number of MPM patients, a substantial number of phase I, phase II, and multicenter (randomized) phase III clinical trials have been undertaken over the last 20 years [7,8]. Despite these clinical trials, pemetrexed/cisplatin has remained the only Food and Drug Administration (FDA)-approved chemotherapy combination, thus illustrating the difficulty in establishing effective therapies for MPM [9]. Chemotherapy has been the focus of MPM research for many years, but in the last 5–10 years, advances in drug development and technologies such as next-generation sequencing have allowed for deeper understanding of the MPM biology and shifted attention to novel (targeted) therapies. This directional change is bearing fruit, as exemplified by a randomized (phase III) study, carried out by a French cooperative group, that observed prolonged survival rates as a consequence of the addition of the antiangiogenic drug bevacizumab to the pemetrexed/cisplatin combination [10] and the FDA approval of immune checkpoint inhibitors, nivolumab and ipilimumab, as first-line therapy for MPM in October 2020 [11,12].

Since the discovery of mesothelin (MSLN) on MPM cells, multiple studies have attempted to exploit this protein as a target for therapy. MSLN is expressed in the majority of epithelioid MPM cases, but it is not expressed in sarcomatoid MPM [5]. MSLN has also been extensively studied as a biomarker in MPM. The aim of this review is to discuss the current status of MSLN as a biomarker and therapeutic target for MPM.

## 2. Mesothelin

MSLN, a glycosylphosphatidylinositol-anchored protein, was discovered almost 30 years ago in an effort to find new surface targets for immunotherapy [13]. It is normally only present in limited amounts on the cell surface of mesothelial cells of the pleura, pericardium, peritoneum, and tunica vaginalis (in men). Our knowledge of the physiological and biological roles of homeostatic MSLN is limited. MSLN does not seem to be required for normal development and reproduction in mice [14,15]. However, MSLN is overexpressed in a number of solid tumors including MPM of the epithelioid histological subtype [16]. MSLN overexpression in several solid tumors such as ovarian, breast, colorectal, and pancreatic cancer has been associated with poor survival rates [17,18,19,20]. However, this association is not clear in MPM. A study of 38 MPM patient tissues suggested that high MSLN expression was correlated with shorter survival rates [21]. However, a larger study with 91 patients found that high MSLN expression was associated with longer survival rates [22], and an even larger study with over 1500 MPM patient tissues reached a similar conclusion [23].

Due to the high overexpression of MSLN in MPM, its role in tumorigenesis has been examined. MSLN is known to bind to mucin 16 (MUC16/CA125), which is expressed by MPM cells and is associated with cancer progression and aggressiveness [24]. The MSLN–MUC16 interaction has been shown to be important for tumor cell adhesion and metastasis, as MSLN can bind MUC16 on other tumor cells [25,26]. MSLN knockdown in MPM results in reduced tumor growth and metastasis in vivo with the downregulation of stem cell and epithelial–mesenchymal-transition (EMT) genes [27]. MSLN expression has been linked to matrix metallopeptidase 9 (MMP-9) expression at the invading edges of tumors, illustrating its role in promoting cancer invasion [28]. Furthermore, MSLN has been implicated in chemoresistance, as the downregulation of MSLN is able to restore cell sensitivity to cisplatin chemotherapy [29].

**Figure 1:**
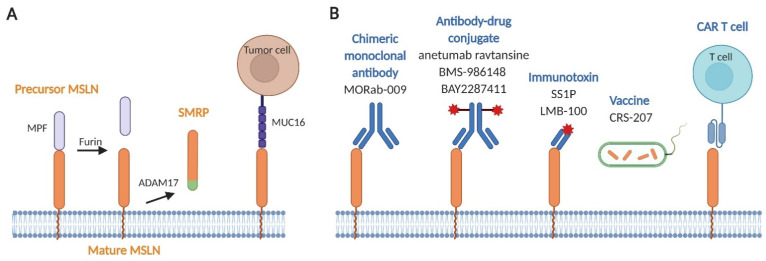
Hitting the bull’s-eye. (**A**) Structural characteristics of mesothelin (MSLN). The MSLN precursor protein (precursor MSLN) at the cell surface is cleaved by furin to release soluble megakaryocyte potentiating factor (MPF), leaving MSLN in its mature form (mature MSLN). Mature MSLN can be shed from the cell membrane by the ADAM17 converting enzyme to form soluble mesothelin-related peptide (SMRP). MPF and SMRP have both been detected in the blood and pleural fluid of MPM patients. MUC16 (or CA125) cells binds to MSLN on tumor for adhesion and to promote cancer metastasis. (**B**) Approaches targeting MSLN used in MPM clinical trials. Several MSLN-targeted therapies have emerged: the chimeric monoclonal antibody MORAb-009; the antibody–drug conjugates anetumab ravtansine, BMS-986148, and BAY2287411; the immunotoxins SS1P and LMB-100; the cancer vaccine CRS-207; and chimeric antigen receptor (CAR) T cell therapy.

MSLN is initially expressed as a precursor glycoprotein of 71 kDa that is cleaved by the endoprotease furin, thus causing a 31 kDa N-terminal soluble protein, called megakaryocyte potentiating factor (MPF), to be released (Figure 1A) [13]. The remaining 40 kDa membrane-bound C-terminal protein is the mature MSLN. This mature form of MSLN can also be shed from the membrane with tumor necrosis factor-α-converting enzyme (TACE; also known as ADAM17), resulting in soluble mesothelin-related peptide (SMRP) [30]. SMRP can be detected in the blood (serum) and pleural fluid of MPM patients and has been the focus of many translational and observational clinical studies, though its biological role is limited [31].

## 3. Mesothelin as a Biomarker

MSLN is by far the most intensively studied biomarker in MPM. MSLN’s derivatives, SMRP and MPF, have been examined as screening, diagnostic, and prognostic biomarkers for MPM [32]. MESOMARK^®^, which detects SMRP, is the only FDA-approved blood test for the management of MPM. Due to the complexity of MPM biology, biomarkers for MPM remain an active area of research [33]. In addition to MSLN, osteopontin and fibulin-3 have also attracted attention as biomarkers for MPM [34,35,36]. High-throughput screens have identified other (potential) biomarkers such as high-mobility group protein 1 (HMGB1), prosaposin, and quiescin Q6 sulfhydryl oxidase 1, as well as epigenetic markers (DNA methylation and miRNAs) [33,37,38,39,40,41,42]. Many of these have been tested in combination with MSLN to improve sensitivity and specificity, but none have so far reached a threshold for routine clinical use.

### 3.1. Diagnosis

The pathological diagnosis of MPM is difficult, and the relatively low prevalence of MPM and microscopic similarities to other cancers contribute to misdiagnoses [43,44,45]. Expert pathologist advice and panels of immunohistochemical markers are frequently needed to reach a correct diagnosis [4,5]. Frequently used markers include mesothelial markers (calretinin or Wilms tumor 1 (WT1)) and carcinoma-related markers (CEA, CD15, Ber-EP4, MOC-31, and TTF-1) [46,47]. Though MSLN is expressed in almost all epithelioid MPM cases, it is also expressed at a significant percentage in adenocarcinomas (particularly in the lung), which results in the failing accuracy of MSLN as a diagnostic marker [16,48,49]. Moreover, MSLN and MPF are negative in the sarcomatoid histological sub-type [50,51].

### 3.2. Screening

The potential to screen for SMRP to detect MPM early has been of much interest for the identification of asbestos-exposed, high-risk individuals. A meta-analysis of the diagnostic value of SMRP in over 4000 patients estimated sensitivity and specificity at 47% and 95%, respectively [31]. Though promising, prospective and retrospective studies in asbestos-exposed risk populations have failed to demonstrate the value of assessing SMRP in blood as a screening tool [52,53,54,55,56,57,58]. Furthermore, there are additional factors that influence SMRP serum levels including age, renal function, genetic background (rs2235503 polymorphism), and body mass index (BMI), which may result in false-positive results if SMRP is used alone [34,54,59,60]. A systematic review of biomarkers in MPM, as part of The European Respiratory Society (ERS)/European Society of Thoracic Surgeons (ESTS)/European Association for Cardio-Thoracic Surgery (EACTS)/European Society for Radiotherapy and Oncology (ESTRO) task force for the evidence-based guidelines in MPM patient management concluded that the routine determination of MSLN and other biomarkers is not supported by the current evidence and that further research into the role of MSLN for diagnosis, screening, and assessing prognosis is still required [2]. Currently, there is only one registered phase I/II clinical trial (NCT04106973) which is examining SMRP levels in asbestos-exposed individuals and its relation to other biomarkers, such as volatile organic compounds from subjects’ breath samples. This trial is currently suspended due to COVID-19-related social distancing restrictions.

### 3.3. Prognosis

It has been suggested that SMRP levels in serum may be associated with prognosis. In a meta-analysis of 8 studies involving 579 MPM patients, high SMRP levels coincided with worse survival rates [61]. However, some studies have been unable to confirm such an association [62,63,64,65,66]. This discrepancy may be caused by the use of different cut-off values (ranging between 1 and 3.5 nmol/L). A recent study suggested that serum MSLN rs1057147 polymorphism in combination with serum SMRP levels might offer better prognostication [63].

### 3.4. Response to Treatment

SMRP assessment in serum has been shown to be helpful for the estimation of tumor response or to predict tumor progression. Serum SMRP levels seem to reflect tumor volume, as patients with large volume tumors have been found to have higher serum SMRP levels [64,67]. In addition, SMRP levels were found to decrease after surgery [30]. Moreover, longitudinal SMRP measurements have corresponded well with tumor response and progression [68,69], and a 10% reduction in serum SMRP level was found to be associated with radiological response [70,71,72], confirming a role for serum SMRP monitoring in MPM patients who initially presented with an elevated SMRP level. Response assessment in patients receiving immunotherapy is occasionally confounded by ‘pseudo-progression’. This phenomenon is caused by immune cells infiltrating the tumor [73] and must be differentiated from tumor progression. SMRP measurements may represent a way to validate whether a lack of tumor shrinkage or an evident increase in tumor size is caused by cellular immune infiltration instead of tumor progression. SMRP levels in MPM seems to be primarily dependent on the histological subtype and not associated with tumor grade [74,75]. Four clinical trials (NCT01265433, NCT02485119, NCT02639091, and NCT02414269) have examined SMRP as a secondary objective in investigating therapies for MPM. One of these studies (NCT02414269), which investigated MSLN-directed chimeric antigen receptor (CAR) T cell therapy, presented preliminary results where decreased serum SMRP levels (<50% compared to pretreatment) were associated with CAR T cell persistence and tumor regression. Data was presented at the American Association for Cancer Research (AACR) 2019 and American Society of Clinical Oncology (ASCO) 2019 conferences.

Similarly to SMRP, MPF (the shed portion generated during the maturation of MSLN) can be detected in the serum samples of MPM patients with a specificity of 90–97% [64,76,77,78]. Increased serum MPF levels were found to be a predictor of poor survival in MPM [79]. Similarly to SMRP, MPF measurements in serum or pleural effusion are not particularly helpful in confirming MPM diagnosis or for screening [76,80]. Combining SMRP and MPF measurements does not affect diagnostic performance, which is likely a consequence of their common origin [78]. It is important to note that the assay used for MPF measurements has not been found to equal the performance of the FDA-approved SMRP assay, MESOMARK^®^ [64,79]. Tumor burden seems to correlate with MPF levels, and reduced MPF levels in the sera of patients receiving anti-MSLN immunotoxin SS1P therapy are associated with improved progression-free and overall survival rates [81]. One clinical trial (NCT03126630), which is currently recruiting, is examining MPF levels. This phase I/II trial is investigating pembrolizumab with and without MSLN-targeted chimeric monoclonal antibody.

MSLN expression represents an important criterion for selecting patients to undergo MSLN-targeted therapy. The used antibody or portion of antibody needs to be carefully considered, as different antibodies that are able to bind to different epitopes of MSLN have been identified. For example, two MSLN antibodies, 5B2 and MN-1, revealed different staining patterns and different rates of positivity [23]. As such, the antibody used to select patients should represent the actual target for therapy. Only a limited number of clinical studies have included the measurement of SMRP or MPF as biomarkers for testing therapies in MPM, and further evidence for their use is required.

## 4. Mesothelin-Targeted Therapies

MSLN has been the focus of immunotherapy research since its discovery as a promising therapeutic target for reducing risk of ‘on-target/off-tumor’ toxicities due to its expression profile in normal and cancer tissue [82,83]. The role of MSLN in promoting tumor invasion and metastatic spread provides another argument to select MSLN as a target [28,84,85,86].

The extracellular domain of MSLN comprises region I (N-terminal region; residues 296–390), II (residues 391–486), and III (C-terminal region; residues 487–598) [87]. Region I correspond to the membrane-distal region (MDR), which binds to MUC16. Due to the role of the MSLN–MUC16 interaction in tumor progression, the MSLN MDR has become the main target for existing immunotherapy strategies [88,89]. However, novel strategies are also targeting other regions to avoid steric hindrance [15,85,90,91,92]. An in vitro study showed that a MSLN-targeted therapy targeting region III had stronger activation and cytotoxicity compared to that targeting region I [92]. This illustrates that the MSLN target region may have an important role in determining the efficacy of MSLN-directed therapies.

Immunotherapy strategies targeting MSLN in MPM include the use of chimeric monoclonal antibody (amatuximab), antibody–drug conjugates (anetumab ravtansine, BMS-986148, and BAY2287411), immunotoxins (SS1P and LMB-100), a cancer vaccine (*Listeria monocytogenes* vaccine expressing MSLN), and CAR T cell immunotherapy (Figure 1B) [89,93]. Table 1 summarizes the clinical trials using these MSLN-targeted therapies against MPM.

### 4.1. Chimeric Monoclonal Antibodies

MORab-009, also known as amatuximab, is a high-affinity monoclonal antibody targeting the MDR region of MSLN. Upon binding, this monoclonal antibody elicits antibody-dependent cellular cytotoxicity (ADCC) and inhibits the adhesion of MSLN-expressing tumor cells to MUC16-expressing tumor cells [94]. In a xenograft mouse model, it was shown to suppress metastasis and enhance the anti-tumor effects of gemcitabine [94,95,96]. Early clinical studies revealed a modest uptake of amatuximab into the pleural tumors of MPM patients [97]. On this basis, as well as on safety data collected in a phase I study [98], amatuximab was investigated in combination with pemetrexed/cisplatin in a single-arm phase II study in patients with unresectable MPM. A promising overall survival rate of 14.8 months and a 90.4% disease control rate (39.8% partial response and 50.6% stable disease; *n* = 83) were reported [99]. In a separate analysis, it was noted that higher amatuximab exposure in combination with chemotherapy was associated with longer overall survival rates, supporting the argument for more frequent dosing [100]. A subsequent randomized, placebo-controlled phase II trial (ARTEMIS, NCT02357147) was prematurely terminated, with no new clinical trials on amatuximab initiated since. It has been noted that amatuximab may bind to MUC16 in the sera of patients, potentially reducing its ADCC activity [99,101,102].

### 4.2. Antibody–Drug Conjugates

An alternative approach is the conjugation of an anti-MSLN antibody with a toxophore, a compound able to produce a toxic effect. In this way, antigen specificity can be combined with toxicity, and tumor cells can be selectively exposed to a tumoricidal agent. Three antibody–drug conjugates (ADCs) have been tested in MPM: anetumab ravtansine, BMS-986148, and BAY2287411.

Anetumab ravtansine (AR) is a human anti-MSLN antibody (MF-T) conjugated to the tubulin inhibitory drug ravtansine (DM4), which disrupts microtubule function [103]. The epitope mapping of the MF-T antibody is still ongoing, but it is known that it binds to a different region than amatuximab. This is supported by the fact that MF-T’s ability to bind MSLN is not affected by MUC16 [104]. In mice models of MPM and ovarian cancer, AR exhibited potent activity against MSLN-positive tumor cells and produced a bystander effect on adjacent MSLN-negative tumor cells [103]. Based on these promising results, the dosage, safety, and efficacy of AR have been tested in phase I and II clinical trials involving MPM patients (NCT02610140, NCT02485119, NCT02696642, NCT01439152, and NCT02639091). In NCT01439152, AR appeared to be safe, with drug-related adverse events in <5% of patients (which were reversible) and no reported drug-related deaths. Regarding efficacy, AR was found to achieve disease control in 75% of MPM patients (5 partial response and 7 stable disease), and high MSLN expression was associated with improved clinical activity [105,106]. This was confirmed in a study using a human uterine xenograft tumor model expressing varying levels of MSLN, which illustrated that AR’s therapeutic response was correlated with the level of MSLN expression in the tumor cells [107]. However, second-line AR tested against vinorelbine in a randomized phase II trial with MPM patients (96% epithelioid subtype) failed to increase progression-free survival rates [108]. To improve clinical efficacy, combinations of AR with other chemotherapies and immunotherapies have been explored. In a phase Ib study (NCT02639091), AR was trialed in combination with standard first-line chemotherapy pemetrexed/cisplatin in MPM and non-small cell lung cancer patients. The study reached an overall response rate of 46% (6 partial response) for AR at the maximum tolerated dose (MTD) for the combination. The toxicity of AR at the MTD was manageable, and no adverse interactions with pemetrexed/cisplatin were observed [109]. A phase I/II trial (NCT03126630) assessing the safety and efficacy of AR in combination with the PD-1 inhibitor pembrolizumab in MPM patients and a phase II trial (NCT03926143) is still recruiting.

BMS-986148 is another anti-MSLN antibody conjugated to a cytotoxic drug, tubulysin. Preliminary data from a phase I/II trial (NCT02341625) with BMS-986148 monotherapy or in combination with nivolumab (anti-PD-1 inhibitor) against MSLN-expressing solid tumors showed that this anti-MSLN ADC was well tolerated (manageable adverse effects). Moreover, some efficacy was reported in MPM patients: 4% overall response rate (ORR) for monotherapy; 31% ORR when given in combination [110]. Durable responses (up to 9 months) were also observed.

BAY2287411 is a thorium-227-labelled antibody-chelator conjugate and the first alpha-particle-emitting therapy. A fully human anti-MSLN antibody is conjugated to thorium-227 via a covalently attached 3,2-HOPO chelator [111]. The emission of alpha particles causes the apoptosis of the target cancer cells by inducing irreversible, double-stranded DNA breaks. Unlike conventional ADC, the prior internalization of the ADC-bound antigen is not required for cytotoxic activity, making this treatment potentially less susceptible to cellular resistance. In MPM xenograft mouse models, BAY2287411 was found to be well-tolerated and showed a high anti-tumor efficacy [111]. Based on these promising preclinical results, a phase I clinical trial (NCT03507452) testing the safety and preliminary activity of BAY2287411 in MPM and ovarian cancer patients is currently in the recruitment phase.

In mouse MPM xenograft models, two different approaches have tested the localized delivery of doxorubicin to MSLN-expressing cancer cells. The first approach used acid-prepared mesoporous silica (APMS) microparticles loaded with doxorubicin and externally modified with an anti-MSLN antibody. Compared to doxorubicin alone, these microparticles showed increased efficacy with fewer adverse side effects [112]. The other approach is based on EnGeneIC Dream Vectors (EDVs). These are bacterial nanocells acting as carriers of cytotoxic drugs or (micro)RNA that can be bound by bispecific antibodies (BsAbs) targeting the EDVs to MSLN. Thus far, MSLN-targeted EDVs carrying doxorubicin have only been tested in mouse MPM xenograft models [113]. EGFR-targeted EDVs carrying a miR-16 mimic (TargomiRs) were tested in a phase I clinical trial in MPM patients who had failed standard chemotherapy. The safety profile of TargomiRs was acceptable, and among 22 patients assessed, 1 partial response and 15 stable disease were observed [114].

### 4.3. Immunotoxins

Recombinant immunotoxins are potent cytotoxic molecules composed of an antibody (or fragment) linked to a bacterial toxin that, once internalized by cancer cells, leads to the inhibition of protein synthesis and apoptosis [115]. The SS1P immunotoxin consists of a high-affinity murine-derived antibody variable fragment (Fv) which binds to the MSLN MDR and is fused to a truncated form of *Pseudomonas* exotoxin A (PE). In mice models, SS1P in combination with gemcitabine was found to achieve strong anti-tumor activity against MSLN-expressing tumors [116]. However, in phase I trials, SS1P resulted in a limited anti-tumor efficacy while clearly exhibiting ‘on-target/off-tumor’ toxicities including the inflammation of the (normal) pleura and the formation of anti-drug antibodies (ADA), which were treated with immunosuppressive drugs [117,118,119]. Therefore, a different formulation of the immunotoxin was explored: LMB-100 [120]. LMB-100 consists of a humanized anti-MSLN Fab fragment (avoiding the formation of ADA) conjugated to a newly designed PE toxin, PE24, that was engineered to be less immunogenic than SS1P. In mice, LMB-100 combined with nab-paclitaxel was well-tolerated and resulted in strong anti-tumor activity [121]. However, this combination was not well-tolerated in a phase I/II study [122], and different combinations and strategies are currently being explored [122,123].

### 4.4. MSLN-Directed Vaccination

Boosting the innate and adaptive immunity of cancer patients is another immunotherapeutic strategy being investigated in MPM. This can be achieved by using MSLN-directed vaccination. One approach is the use of the *Listeria monocytogenes* vaccine expressing MSLN (LM-mesothelin), CRS-207. This cancer vaccine builds on the immunogenicity of a genetically modified *Listeria monocytogenes* to boost immunity against MSLN-expressing cancer cells [124]. After showing a good safety profile in a phase I study (NCT00585845) [125], CRS-207 was tested as monotherapy and in combination with cyclophosphamide. In combination with cyclophosphamide, the vaccine showed no unexpected toxicities; 89% of the 35 patients evaluated had disease control, with 1 patient achieving complete response (19 partial responses and 10 stable disease). In addition, combination therapy induced positive changes in the tissue microenvironment, with the reinvigoration of the immune response and objective tumor responses in the majority of patients [126].

Other approaches aimed at promoting dendritic cell maturation, T cell activation, and (consequently) anti-tumor immunity have been tested in mouse MPM xenograft models. These include a chimeric DNA vaccine using a MSLN-specific connective tissue growth factor (CTGF/MSLN) combined with immune-modulators [127] and a novel immunotherapeutic agent consisting of an anti-MSLN single-chain variable fragment (scFv) bound to the heat shock protein 70 (Hsp-70) from *Mycobacterium tuberculosis,* which acts as immune activator [128]. Both immunotherapeutic strategies demonstrated promising results, with enhanced MSLN-specific anti-tumor immunity and increased survival rates.

### 4.5. Mesothelin-Targeted Cellular Therapy

Adoptive cell therapy targeting MSLN has emerged as a potential therapy for the treatment of MPM with numerous clinical trials [129]. Chimeric Antigen Receptor (CAR) T cellular immunotherapy entails genetically engineering T cells to target the tumor. The CAR structure has evolved over the years, building on the success of CD19-directed CAR T cell therapies in hematological malignancies and leading to their FDA approval [130]. Typical features include an ectodomain, containing the single-chain variable fragment (scFv) that identifies and binds to a specific tumor antigen (in this case, MSLN), a hinge, a transmembrane domain, and an endodomain that contains the signaling domains. The evolving endodomain structures have given rise to several different CAR generations that typically consists of CD3ζ and/or co-stimulatory elements such as 4-1BB, CD28, OX40, or ICOS [131]. Compared to other MSLN-targeted therapies, CAR T cell therapy offers the potential to promote immune surveillance and avoid tumor recurrence through CAR T cell persistence in the patients’ body and then reactivation after a further antigen encounter [132]. Anti-MSLN CAR T cells have also been shown not to react with SMRP [133] and only initiate their cytotoxic activity against membrane-bound MSLN [134,135].

Promising preclinical studies demonstrated that CAR T cells with the SS1 scFv eradicated established MSLN-positive tumors with no toxicity [135,136]. This led to two phase I trials (NCT01355965 and NCT02159716) in MPM. The first achieved a satisfactory safety profile with transient tumor responses noted in 2 of 18 patients [137]. The second resulted in stable disease in 11 (out of 15) patients at day 28 post-infusion, but 5 of these progressed at a later time point [138]. The limited efficacy may have been due to the observed low persistence and low tumor infiltration of the CAR T cells. A subsequent trial by the same group is currently recruiting and will administer CAR T cells through two different routes: intravenously and locoregionally directly into the pleural space (NCT03054298).

Locoregional delivery is used to improve efficacy and persistence of CAR T cells. The goal of locoregional delivery is to circumvent the observed lack of trafficking and homing to the MPM tumor (overcome the barriers of tumor stroma). This has also been addressed by the further genetic modification of anti-MSLN CAR T cells to express chemokine receptor CCR2 [139] and by combination with a CXCR4 antagonist [140]. In preclinical studies, intrapleural delivery has resulted in more effective tumor reduction and increase in survival rates. It was also found that it required a 30-fold lower dose for successful tumor eradication compared to intravenous injection, suggesting the potential for a lower CAR T cell dosage in patients [134]. This has led to a number of trials administering CAR T cells locoregionally (NCT03608618, NCT02414269, NCT03054298, and NCT04577326). Results from NCT02414269 demonstrated that 2 (out of 16) patients had partial response, 9 had stable disease, and 5 had progressive disease. No major toxicities were reported in these patients. Interestingly, this trial also tested CAR T cells in combination with an anti-PD-1 inhibitor, pembrolizumab, and results suggest that there may be synergy and clinical benefit in combining CAR T cells with immune checkpoint inhibitors to overcome CAR T cell exhaustion and prolong functional persistence [141,142]. This strategy is also being tested in another clinical trial (NCT03907852) that uses a different type of T cell genetic modification to recognize MSLN, which is called TCR fusion Construct (TRuC). Preliminary results presented at AACR 2021 were very encouraging and showed tumor regression in all 8 treated patients [143].

Rather than combining immune checkpoint inhibitors with CAR T cells and being confronted again with ‘trafficking’ issues, CAR T cells can be engineered to carry a PD-1 dominant negative receptor (NCT04577326) [144] or modified to secrete anti-PD-1 nanobodies (NCT04489862). The two trials investigating this approach are still in the recruitment phase, so the advantage of these strategies is not yet known.

Overall, the results of MSLN-directed CAR T cell therapy are encouraging, with manageable toxicity and no reported ‘on-target/off-tumor’ effects. Novel strategies to improve CAR T cell efficacy are currently being tested in clinical trials, with many more in the preclinical stage [129]. Furthermore, allogeneic ‘off the shelf’ MSLN-targeted CAR T cells are being developed. They are yet to be tested in the clinical setting for MPM, but preclinical studies have shown encouraging results so far [145,146]. Another approach not yet tried against MPM is the genetic modification with an anti-MSLN CAR of other types of immune cells such as macrophages [147] and natural killer cells [148,149]. Anti-MSLN CAR natural killer cell therapy is currently being tested against ovarian cancer in a phase I clinical trial (NCT03692637).

## 5. Conclusions

MSLN is highly expressed in MPM but only modestly expressed in normal tissue, making it a promising potential biomarker and therapeutic target. MSLN has been implicated in key tumorigenic roles such as MPM tumor growth, metastasis, and drug resistance, but its exact mechanisms are poorly understood. A substantial number of clinical trials have investigated MSLN as a biomarker or as a therapeutic target in MPM. Soluble MSLN (SMRP) measured from serum samples has a high specificity but lacks sensitivity for MPM diagnosis, screening, and prognosis; however, it is a useful biomarker to monitor response to therapy because its expression is associated with tumor volume.

Several MSLN-targeted immunotherapies, including chimeric monoclonal antibodies, antibody–drug conjugates, immunotoxins, vaccines, and CAR T cells, have been developed. Results from clinical trials using these immunotherapeutic strategies have demonstrated no major on-target/off-tumor toxicities, and therapies have been generally well-tolerated. The large majority of MPM cases overexpress MSLN. Therefore, MSLN-targeted immunotherapies may represent a well-directed approach for the majority of patients presenting with epithelioid MPM. Thus far, clinical responses with MSLN-directed immunotherapies have been modest, so strategies are needed to enhance the therapeutic efficacy while maintaining a favorable toxicity profile in order to improve the poor outlook of MPM patients. Some of these enhancement strategies and drug combinations are already in the clinical trial stage, especially CAR T cell therapies, and raise hope for increased efficacy.

## Figures and Tables

**Table 1:** MSLN-targeted immunotherapies in MPM.

Clinicaltrials.gov Identifier	Phase	Intervention	Cancer Type	Sponsors and Locations	Status	Outcomes
**Chimeric monoclonal antibodies**						
**Amatuximab (MORAB-009)**						
NCT01018784	I	MORAb-009	MPM, colorectal, pancreatic, and head and neck cancers	Eisai Co., Ltd., Japan	Completed (February 2013)	Efficacy: 3/17 SD (for 47–217 days). Safety: Treatment well-tolerated up to 200 mg/m^2^.
NCT01521325	I	Indium-111-labelled MORAb-009	MPM, pancreatic, ovarian, and NSCLC	Morphotek and National Cancer Institute, USA	Completed (March 2013)	Safety: well-tolerated with favorable dosimetry profile. Radio-labelled MORAb-009 demonstrated higher uptake in MM than pancreatic cancer and bound to both primary and metastatic sites.
NCT01413451	I	Indium-111-labelled MORAb-009	MPM, ovarian, and NSCLC	National Institutes of Health Clinical Center, USA	Terminated (November 2013)	Safety: well-tolerated with favorable dosimetry profile.
NCT00738582	II	MORAb-009 with pemetrexed and cisplatin	Unresectable MPM	Morphotek in Canada, Germany, Netherlands, Spain, USA	Completed (January 2014)	OR: 33/83 PR, 42/83 SD. PFS: 6.1 months, OS: 14.8 months. Safety: meutropenia (15/83) and anemia (9/83) were the most common grade 3 and 4 AE. Treatment generally well-tolerated.
NCT02357147	II	MORAb-009 with pemetrexed and cisplatin	Unresectable MPM	Morphotek in Australia, France, Germany, Italy, UK, USA	Terminated (November 2018)	Safety: treatment was generally well-tolerated.
**Antibody–drug conjugates**						
**Anetumab ravtansine (BAY94-9343)**						
NCT02610140	II	Anetumab ravtansine or vinorelbine	Advanced/metastatic MPM	Bayer, collaborating with ImmunoGen and MorphoSys in Australia, Belgium, Canada, Finland, France, Italy, South Korea, Netherlands, Poland, Russian Federation, Spain, Turkey, UK	Completed (May 2017)	Similar PFS of 4.3–4.5 months and OS of 9.5 months for anetumab ravtansine compared to 11.6 months for vinorelbine, 8.4 vs. 6.1% OR, and 34 vs. 35% serious adverse events.
NCT02485119	I	Anetumab ravtansine	Advanced malignancies including MPM	Bayer in Japan	Completed (July 2017)	Safety: treatment was generally well-tolerated.
NCT02696642	I	Anetumab ravtansine	Predominantly epithelial (>50% of tumor composition) pleural/peritoneal MM and other MSLN+ solid tumors w/wo renal or hepatic impairments	Bayer in France, Republic of Moldova	Completed (July 2019)	Results not published.
NCT01439152	I	Anetumab ravtansine	Epithelial peritoneal MM, advanced MPM, and platinum-resistant ovarian cancer	Bayer in USA	Completed (July 2019)	Efficacy: 1/138 CR, 11/138 PR, 66/138 SD, median PFS = 2.8 months. Safety: no drug-related deaths. All drug-related AE (≥5% of all participants) were reversible.
NCT02639091	Ib	Anetumab ravtansine with pemetrexed and cisplatin	Epithelial pleural/peritoneal MM, NSCLC	Bayer in USA and Italy	Completed (October 2019)	OR: 8/16 PR, set MTD to 6.5 mg/kg.
NCT03126630	I/II	Pembrolizumab w/wo anetumab ravtansine	MPM	National Cancer Institute, USA and Canada	Recruiting	
NCT03926143	II	Anetumab-ravtansine (continued treatment)	Solid tumors previously treated with anetumab-ravtansine	Bayer in USA, France, Italy, Poland.	Recruiting	
**BMS-986148**						
NCT02884726	I	BMS-986148	MPM and other advanced solid tumors	Bristol-Myers Squibb in Japan	Completed (September 2017)	Results not published.
NCT02341625	I/IIa	BMS-986148 w/wo nivolumab	Advanced MPM, ovarian, pancreatic, gastric, and NSCLC	Bristol-Myers Squibb in Australia, Belgium, Canada, Italy, Netherlands, UK, USA	Active, not recruiting	Preliminary results (April 2019) showed the best OR of 31%. Durable responses in MPM patients ~9 months. 44% of participants (*n* = 126) developed grade 3/4 treatment-related AE. 1 death due to treatment-related pneumonitis reported.
**BAY2287411**						
NCT03507452	I	BAY2287411 (thorium-227-labelled antibody-chelator conjugate)	Advanced recurrent epithelioid MM, ovarian cancer, and PDAC	Bayer in USA, UK, Sweden, Netherlands, Finland	Recruiting	
**Immunotoxins**						
**SS1P**						
NCT00066651	I	Immunotoxin SS1P bolus infusion	MPM, cervical, fallopian tube, head and neck, lung, ovarian, pancreatic, and primary peritoneal cavity cancers	Warren Grant Magnuson Clinical Center—NCI Clinical Studies Support, Comprehensive Cancer Center at Wake Forest University, USA	Completed	Safety: grade 3 pleuritis at highest DLT. No grade 4 toxicities; well-tolerated. Efficacy: 4/33 minor responses, 19/33 SD, 19/33 PD.
NCT00006981	I	Immunotoxin SS1P continuous infusion	MPM, cervical, fallopian tube, head and neck, lung, ovarian, PDAC, and primary peritoneal cavity cancers	Warren Grant Magnuson Clinical Center—NCI Clinical Studies Support, USA	Completed	Efficacy: 1/24 PR (ovarian), 12/24 SD, 11/24 PD. Safety: grade 4 acidosis. Participants with existing pulmonary hypertension and diastolic dysfunctions developed large pleural effusions and respiratory failure upon treatment. Generally well-tolerated. Overall, continuous infusion was not better than bolus dosing.
NCT01445392	I	Immunotoxin SS1P (single or multicycle) with pemetrexed and cisplatin	MPM	National Institutes of Health Clinical Center, USA	Terminated (October 2016)	Efficacy: 12 PR, 3 SD, and 5 PD out of 20 evaluable participants. 10 PR, 1 SD, 2 PD out of 13 patients who received MTD. Safety: no treatment-related grade 4 AE and 1 death due to neutropenic sepsis during treatment, likely due to underlying chronic kidney disease.
NCT01362790	I/II	Immunotoxin SS1P with pentostatin and cyclosporamide	MPM, lung, and PDAC	National Institutes of Health Clinical Center, USA	Completed (August 2017)	OS from 4.2 to 29.3 months, PFS from 3.9 to 11.8 months depending on different dose regimens, 2/55 PR and 24/55 SD. Safety: no grade 4 AE attributed to SSP1. Pentostatin or cyclophosphamide caused grade 4 lymphopenia.
**LMB-100**						
NCT03436732	I	Immunotoxin LMB-100 with SEL-110 (biodegradable nanoparticle containing rapamycin for preventing anti-drug-antibodies formation)	MPM	National Institutes of Health Clinical Center, USA	Terminated (April 2019)	Terminated before primary outcomes due to 1 case of pneumonitis associated with drugs in the 100 mcg/kg LMB-100 and SEL-110 arm.
NCT03644550	II	Immunotoxin LMB-100 followed by pembrolizumab	MPM	National Institutes of Health Clinical Center, USA	Completed (November 2020)	Preliminary results (April 2018) showed an efficacy of 1 CR, 3 PR, 1 SD, and 2 PD (*n* = 7). With checkpoint inhibitor: median OS was 11.9 months.
NCT02798536	I	Immunotoxin LMB-100 w/wo nab-paclitaxel (Abraxane)	MPM	National Institutes of Health Clinical Center, USA	Completed (January 2021)	Positive results for safety.
NCT04840615	I	Intratumoral injection of Immunotoxin LMB-100 with ipilimumab	MPM	National Institutes of Health Clinical Center, USA	Recruiting	
NCT04034238	I	Immunotoxin LMB-100 combined with tofacitinib	Epithelioid MPM, extrahepatic cholangiocarcinoma, and PDAC	National Institutes of Health Clinical Center, USA	Recruiting	
NCT02810418	I/II	LMB-100 (via short or continuous infusion) w/wo nab-paclitaxel	Previously treated mesothelin-expressing solid tumors including MPM	National Institutes of Health Clinical Center, USA	Active, not recruiting	Preliminary results (May 2020): no objective responses (*n* = 15). Long infusion of LMB-100 was well-tolerated but led to higher immunogenicity (i.e., higher titers of anti-drug antibodies). Updated preliminary results (February 2021) showed the best OR for short infusion as: 1/14 PR. PFS not presented due to patients receiving other treatments afterwards. 4 cases of grade 4 LMB-100-associated AE (*n* = 40).
**Cancer vaccines**						
**CRS-207**						
NCT00585845	I	CRS-207	Epithelial MPM, ovarian, pancreatic, and NSCLC cancers which failed standard treatments	Anza Therapeutics, Inc. in USA and Israel	Terminated (February 2009)	Efficacy: 6/17 survived for ≥15 months (1 mesothelioma participant). Safety: well-tolerated. MTD set to 1 × 10^9^ cfu. No grade 5 AE observed, only transient grade 4 lymphopenia. Above MTD (1 × 10^10^ cfu): 1 case of grade 2 CRS.
NCT03175172	II	CRS-207 with pembrolizumab	MPM epithelial/biphasic	Aduro Biotech, Inc., collaborating with Merck Sharp and Dohme Corp., USA	Terminated (January 2018)	OR: 1/9 SD, PFS: 3.4–8.9 weeks, and 4/10 AE (grade not reported). Terminated due to low enrolment and lack of clinical activity.
NCT02575807	I/II	CRS-207 w/wo epacadostat (an IDO1 inhibitor) and/or pembrolizumab	Platinum-resistant peritoneal, ovarian, and fallopian cancers	Aduro Biotech Inc., collaborating with Incyte Corporation in USA and Canada	Completed (May 2018)	OR: 6/32 SD, OS: 4.71–88.71 weeks. Safety: 23/28 with grade 3 or higher AE. Terminated due to low enrolment and lack of clinical response.
NCT01675765	Ib	CRS-207 w/wo cyclophosphamide followed by standard-of-care chemotherapy (pemetrexed and cisplatin)	MPM	Aduro Biotech, Inc. in USA	Completed (September 2018)	Treatment without cyclophosphamide had slightly better responses (2.8 vs. 0% CR, 53 vs. 52% PR, 39 vs. 38% SD, and 36 vs. 21 total participants), less AE (39 vs. 50%) but higher all-cause mortality (8 vs. 0%).
**CAR T cell therapies**						
NCT01355965	I	mRNA anti-MSLN CAR-T cells	MPM and PDAC	University of Pennsylvania, USA	Completed (October 2015)	Positive results (primary endpoint: safety). Best OR: 2/18 patient showed transient response.
NCT02159716	I	Anti-MSLN CAR-T cells (CART-meso)	MPM, PDAC, and ovarian	University of Pennsylvania, USA	Completed (November 2015)	Positive results (primary endpoint: safety). Low persistence and low tumor infiltration were observed. Best OR: 6/15 patients with SD.
NCT01583686	I/II	Anti-MSLN CAR-transduced peripheral blood lymphocytes with fludarabine, cyclophosphamide, and IL-2	MSLN-expressing tumors	National Institutes of Health Clinical Center, USA	Terminated due to slow/insufficient accrual (December 2018)	Positive results for safety but low efficacy. Best OR: 1/15 with SD. 14/15 with PD.
NCT03054298	I	Anti-MSLN CAR-T cells (huCART-meso) with cyclophosphamide	MSLN-expressing tumors including MPM	University of Pennsylvania, USA	Recruiting	
NCT03608618	I	mRNA anti-MSLN CAR PBMC (MCY-M11) with cyclophosphamide	Peritoneal MM, fallopian tube and ovary adenocarcinoma, and primary peritoneal carcinoma	MaxCyte, Inc., USA	Active, not recruiting	Preliminary results (ASCO 2020): positive results on safety. 4/11 patients with SD.
NCT02414269	I/II	Anti-MSLN CAR-T cells with suicide switch (iCasp9M28z) and cyclophosphamide or pembrolizumab	MPM	Memorial Sloan Kettering Cancer Center, USA	Recruiting	Positive results on safety: no DLT, no grade 5 AE and manageable Grade 4 AE. Median OS was 17.7 months and 1-year OS was 74%. In cohort treated with pembrolizumab median OS was 23.9 months and 1-year OS was 83%. Best OR was 2/16 patients with PR, 9/16 with SD and 5/16 with PD based on mRECIST criteria.
NCT04577326	I	Anti-MSLN CAR T cells with intrinsic anti-PD1 inhibition (M28z1XXPD1DNR and ATA2271) and cyclophosphamide	MPM	Memorial Sloan Kettering Cancer Center in collaboration with Atara Biotherapeutics, USA	Recruiting	
NCT03907852	I/II	Anti-MSLN TCR fusion Construct (TRuC) T cells (gavo-cel and TC-210) with cyclophosphamide, pembrolizumab, and fludarabine	MSLN-expressing tumors	TCR^2^ Therapeutics, USA and Canada	Recruiting	Preliminary results (AACR 2021): single gavo-cel infusion was generally safe and resulted in tumor regression in all 8 patients treated (disease control rate: 100%) and objective responses in 3 (2 with MPM and 1 with ovarian cancer). Addition of lymphodepletion resulted in higher gavo-cel peak expansion, associated with greater tumor regression and objective responses.
NCT04489862	I	Anti-MSLN CAR-T cells expressing PD-1 nanobodies	MPM and NSCLC	Wuhan Union Hospital, China	Recruiting	
NCT03638206	I/II	Anti-MSLN CAR-T cells with cyclophosphamide and fludarabine	MPM	Shenzhen BinDeBio Ltd., China	Recruiting	

**Abbreviations**: MSLN = mesothelin; MM = malignant mesothelioma; MPM = malignant pleural mesothelioma; USA = United States of America; UK = United Kingdom; PDAC = pancreatic ductal adenocarcinoma; NSCLC = non-small cell lung cancer; OR = overall response; OS = overall survival; SD = stable disease; PD = progressive disease; PR = partial response; CR = complete response; AE = adverse effects; PFS = progression-free survival; MTD = maximum tolerated dose; DLT = dose-limiting toxicity; PBMC = peripheral blood mononuclear cells; CAR = chimeric antigen receptor; PD-1 = programmed cell death protein 1; DNR = dominant negative receptor; ASCO = American Society of Clinical Oncology; mRECIST = modified Response Evaluation Criteria in Solid Tumors; AACR = American Association for Cancer Research.

## Data Availability

Data sharing is not applicable to this article. No new data were created or analyzed in this study.
